# A Massive Type IV Hiatal Hernia With Gastric Volvulus and the Complicated Road to Recovery

**DOI:** 10.7759/cureus.108043

**Published:** 2026-04-30

**Authors:** Spencer Barnes, Abel Abraham, Anjelli Wignakumar, Sasha Rackman

**Affiliations:** 1 College of Medicine, Florida Atlantic University Charles E. Schmidt College of Medicine, Boca Raton, USA; 2 Colorectal Surgery, Cleveland Clinic, Miami, USA; 3 Geriatrics, Florida Atlantic University Charles E. Schmidt College of Medicine, Boca Raton, USA

**Keywords:** biological mesh, geriatric surgery, non-diabetic gastroparesis, paraesophageal hiatal hernia, robotic hiatal hernia

## Abstract

Type IV paraesophageal hiatal hernias are rare but carry a significant risk of life-threatening complications, including gastric volvulus, incarceration, and the involvement of other intra-abdominal organs. Pancreatic herniation and associated pancreatitis are exceedingly uncommon. We report the case of a 74-year-old woman with Barrett’s esophagus, chronic obstructive pulmonary disease (COPD), and obesity who presented with nausea, vomiting, and abdominal pain after a recent increase in the dose of tirzepatide. Imaging demonstrated a massive type IV paraesophageal hernia with organoaxial gastric volvulus. Initial laparoscopic reduction was aborted due to dense adhesions and a foreshortened esophagus. She subsequently developed progressive herniation involving the stomach, colon, and pancreatic body and tail, and ultimately underwent delayed robotic-assisted repair.

The patient's postoperative course was complicated by multiple recurrences, including episodes of volvulus, duodenal obstruction, and mild pancreatitis. She ultimately required open repair with mesh reinforcement and gastropexy after unsuccessful endoscopic detorsion. Despite temporary improvement, she later developed recurrent herniation and severe gastroparesis, likely secondary to injury to the vagal nerve. This report highlights the complexity of managing massive type IV paraesophageal hernias, particularly with multi-organ involvement and recurrence. Even with advanced surgical approaches, recurrence and long-term complications remain significant challenges.

## Introduction

Paraesophageal hiatal hernias are categorized into types 2, 3, and 4, with severity increasing according to the numerical classification. Type 4 paraesophageal hernias represent the rarest form of hiatal hernia and account for approximately 5% of cases [1]. Potential complications of type 4 hiatal hernias include intrathoracic incarceration of the stomach, bleeding, perforation, and gastric volvulus [2]. We describe the initial presentation and sequelae of an older woman who presented with a type 4 hiatal hernia, including parts of the large intestine, small intestine, mesentery, and pancreatic tail.

## Case presentation

A 74-year-old female with a past medical history of Barrett’s esophagus, chronic obstructive pulmonary disease (COPD), dyslipidemia, obesity, and breast cancer status post lumpectomy presented to the ED with nausea, vomiting, abdominal pain, and headache. The patient’s Zepbound dose had been increased to 2 mg two days before the ED visit. The patient denied diarrhea and stated that her last bowel movement had been that morning. She also reported significantly increased belching compared to normal. Regular medications included albuterol as needed, anastrozole, esomeprazole, metformin, simvastatin, and Trelegy Ellipta. Vital signs were normal except for a BMI of 31 kg/m^2^. The physical exam performed in the ED was significant for a soft abdomen with mild mid-abdominal tenderness and normal bowel sounds. Labs were significant for leukocytosis at 12.2 K/mcL and thrombocytosis at 416 K/mcL.

**Figure 1:**
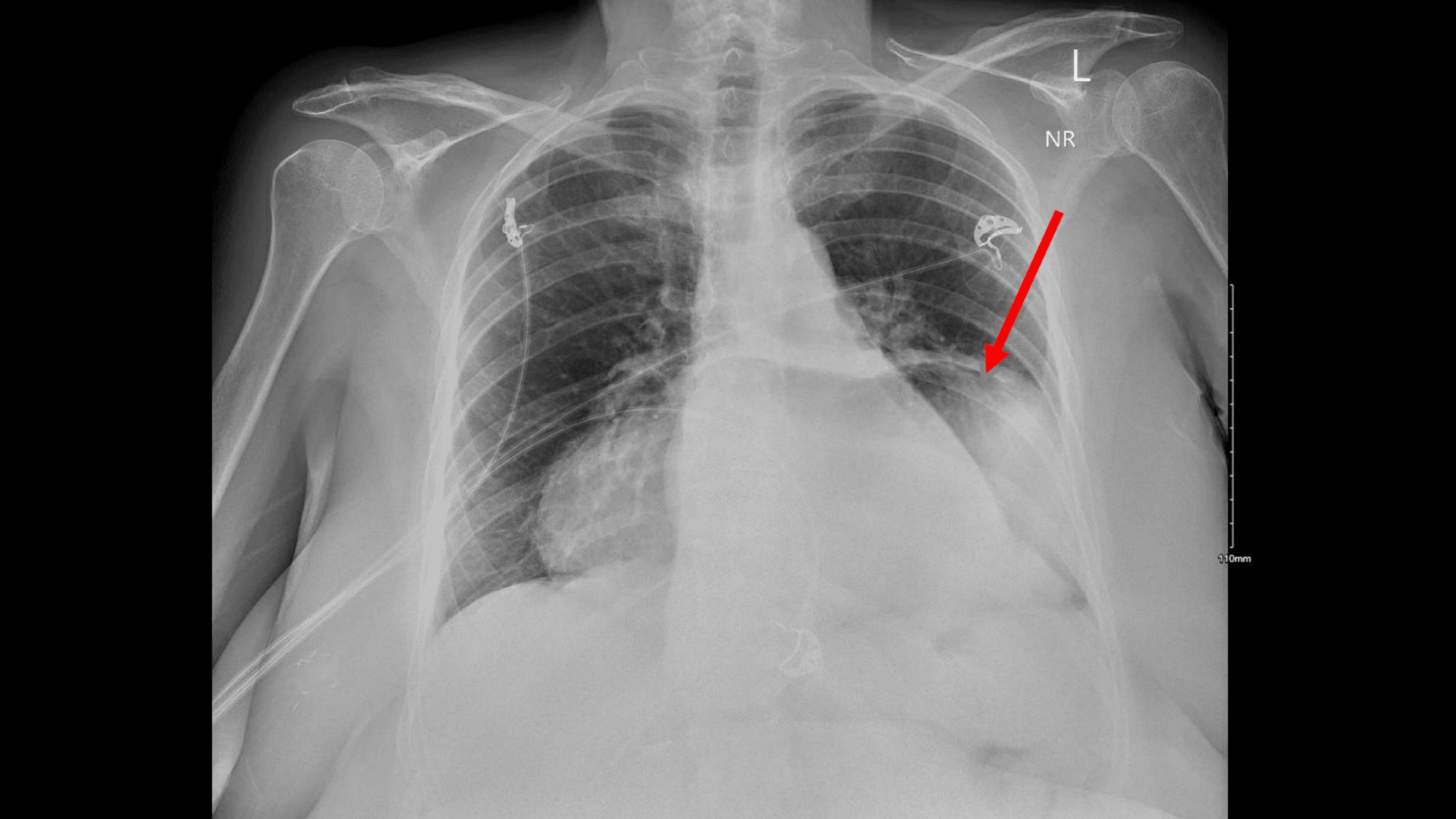
Bedside chest X-ray taken on hospital day 1 Findings: a large diaphragmatic/gastric hernia with distention of the stomach

**Figure 2:**
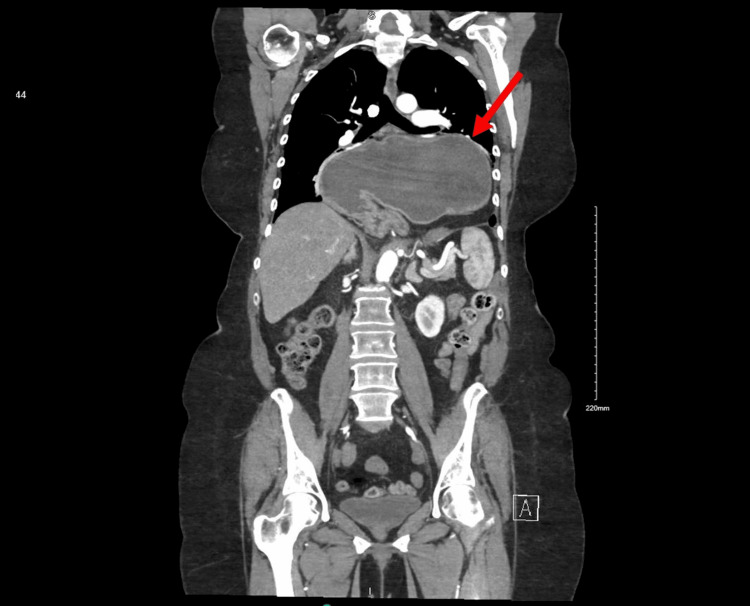
CT angiography of the chest taken on hospital day 1 The image demonstrated a very large posterior diaphragmatic hernia with the stomach extending into the mid chest level. An organoaxial volvulus with the duodenum located inferiorly was also identified CT: computed tomography

The patient’s abdominal pain resolved with appropriate rehydration and antiemetics, and she was tolerating oral intake before discharge. Approximately six hours after discharge, the patient returned to the ED due to worsening substernal chest pain. An EKG was performed and was deemed noncontributory. A bedside chest X-ray revealed a large diaphragmatic/gastric hernia with distention of the stomach (Figure 1). CT angiography of the chest (Figure 2) demonstrated a very large posterior diaphragmatic hernia with the stomach extending into the mid-chest level. An organoaxial volvulus, with the duodenum located inferiorly, was also identified. An esophagogastroduodenoscopy (EGD) was performed later that day, which revealed a large amount of solid and liquid food in the stomach and distal esophagus. The majority of the food was cleared from the distal esophagus, and the gastric mucosa that was visible appeared normal. A nasogastric (NG) tube was placed and set to low intermittent suction.

Laparoscopic evaluation and reduction of the hiatal hernia with EGD were performed. The large hernia defect was noted to have much of the stomach in the thoracic cavity, and a significant amount of omentum was reduced from the chest to allow visualization of the stomach. Extensive adhesions were documented at the apex of the chest cavity, which impeded stomach reduction. This surgical approach was not sufficient due to the significant scarring in the upper esophagus, in addition to the esophagus itself being markedly foreshortened. The operation was aborted, and the patient was scheduled to follow up as an outpatient with the surgical team to further explore surgical treatment options. Four days later, the patient returned to the ED complaining of chest and abdominal pain. CT again demonstrated the large hiatal hernia, with paraesophageal hernia components noted to include mesenteric fat and segments of the transverse colon. Portions of the pancreatic body and tail were noted on CT. The patient began feeling better and followed up with cardiothoracic surgery a week later.

Approximately six weeks after the initial presentation, the cardiothoracic surgeon performed a robotic-assisted hiatal hernia reduction and repair without mesh. After placing ports, the colon and stomach were visualized in the chest cavity. The colon was retracted and reduced into the abdomen. Hernia sac dissection was initiated at the 9 o'clock position and extended over the superior aspect to the 3 o'clock position, and the sac was mobilized throughout the thoracic cavity, mobilizing the stomach and esophagus. Extensive dissection of the hernia sac from the mediastinum was performed, and this assisted in lengthening the esophagus approximately 3 cm below the diaphragm. The remaining dissection followed the crus of the diaphragm on the left and right. The entire stomach and esophagus were subsequently mobilized. A 46 French bougie was placed into the esophagus, and the posterior aspect of the crus was closed with approximately six interrupted 0 silk sutures. There was no evidence of retraction of the stomach into the thoracic cavity. A chest X-ray was performed to confirm the reduction of the hernia, and a follow-up X-ray 10 days after surgery revealed postoperative improvement.

**Figure 3:**
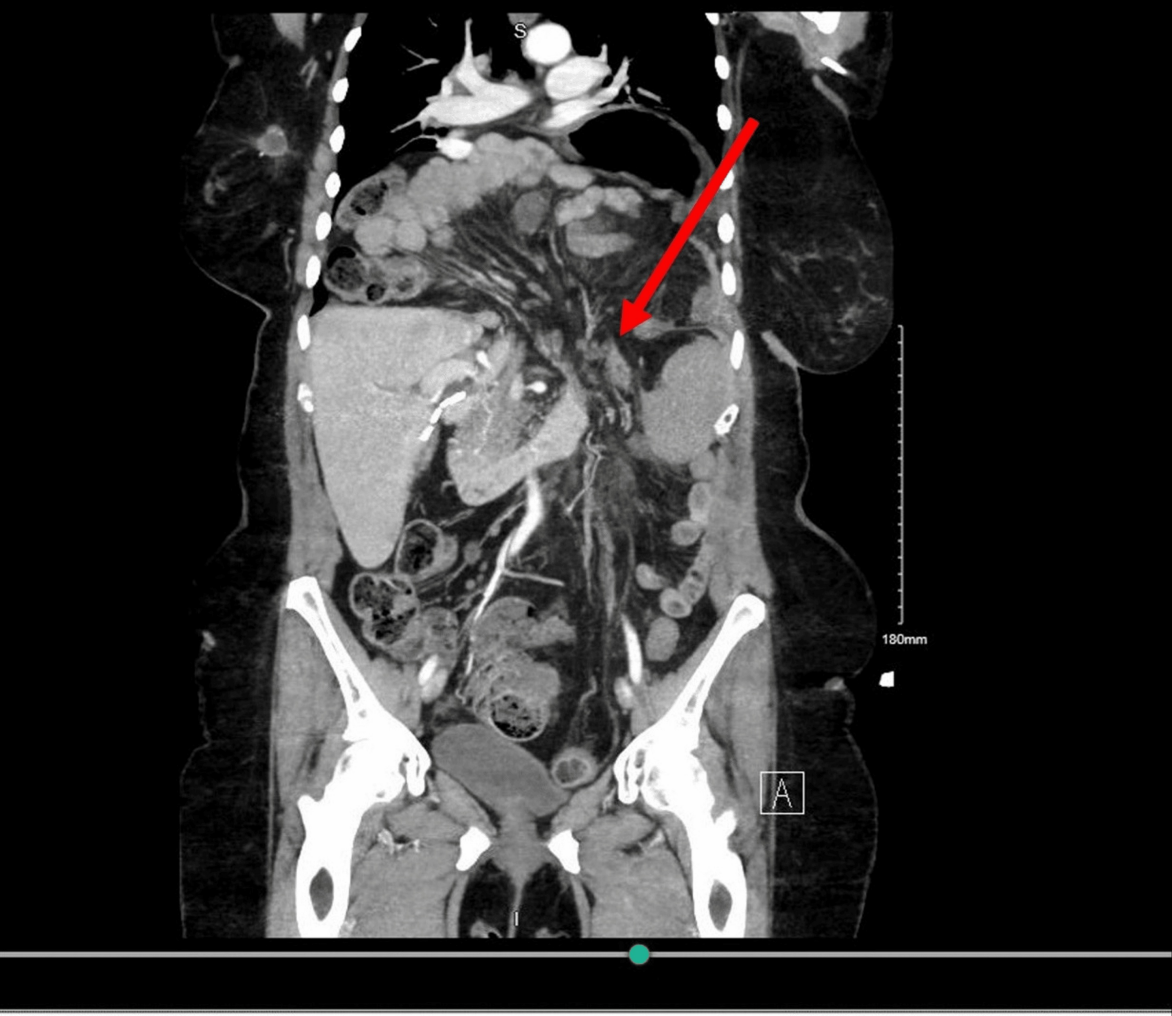
CT taken on readmission day 1 - image 1 The image demonstrated a recurrence of the hiatal hernia containing the majority of the stomach, large portions of small bowel, and transverse colon. Edema was imaged around the pancreatic tail, which was reflective of mild pancreatitis CT: computed tomography

**Figure 4:**
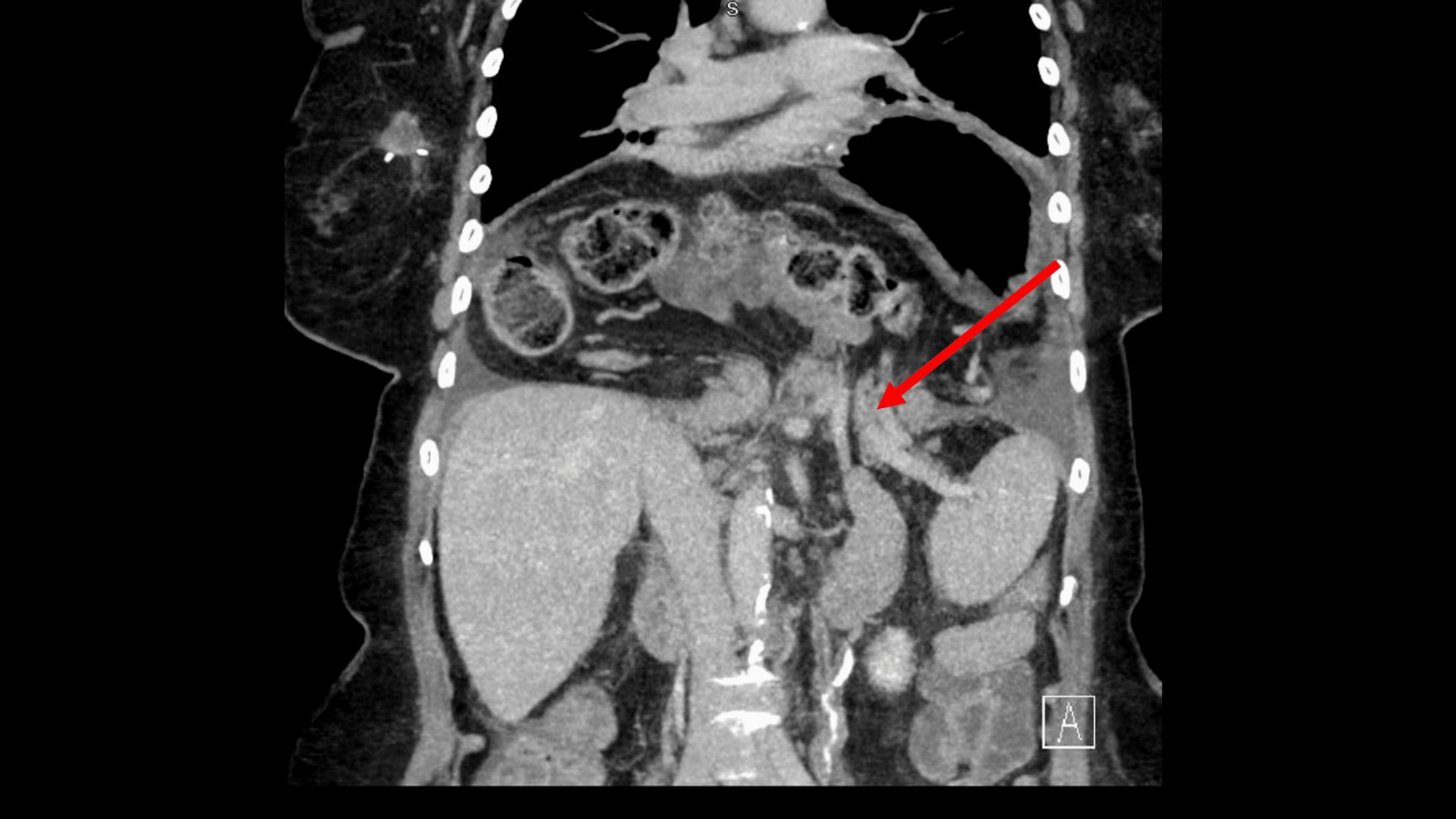
CT taken on readmission day 1 - image 2 The image demonstrated a recurrence of the hiatal hernia containing the majority of the stomach, large portions of small bowel, and transverse colon. Edema was imaged around the pancreatic tail, which was reflective of mild pancreatitis CT: computed tomography

Almost three months later, the patient presented to the ED with abdominal pain and vomiting. At that time, labs were significant for elevated amylase (140 IU/L) and lipase (511 U/L). CT (Figures 3, 4) demonstrated a recurrence of the hiatal hernia containing the majority of the stomach, large portions of the small bowel, and the transverse colon. Edema was noted around the pancreatic tail, which was consistent with mild pancreatitis. Supportive care was provided in the hospital, and repeat CT and ultrasound were performed to confirm recovery. The consulting cardiothoracic surgeon did not feel that surgical intervention was indicated, and the patient was discharged. 

**Figure 5:**
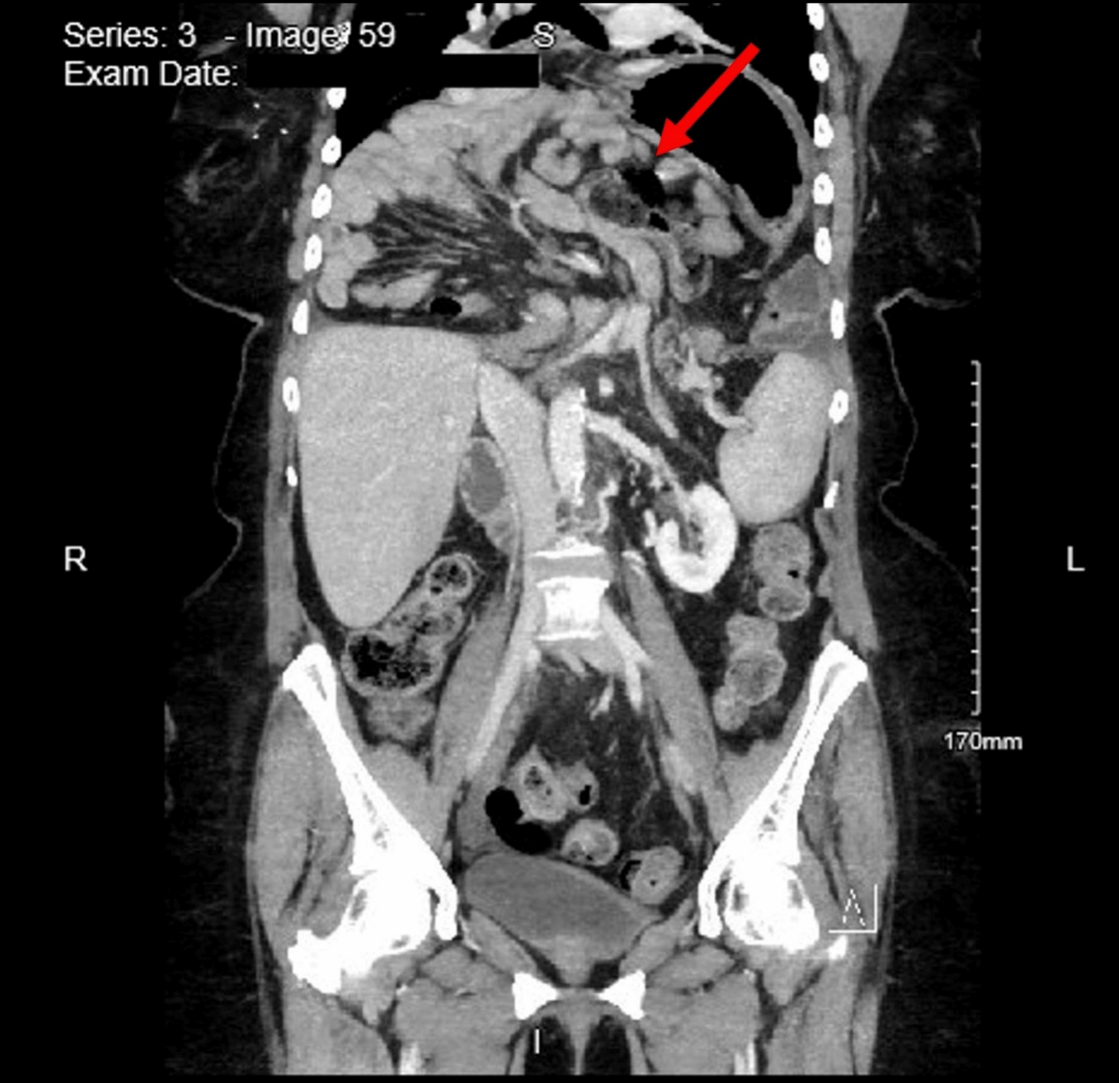
CT taken 4 months after the initial presentation The image showed a very large hiatal hernia containing the stomach, some small bowel loops, and a portion of the transverse colon. Some surrounding ascites were also observed CT: computed tomography

**Figure 6:**
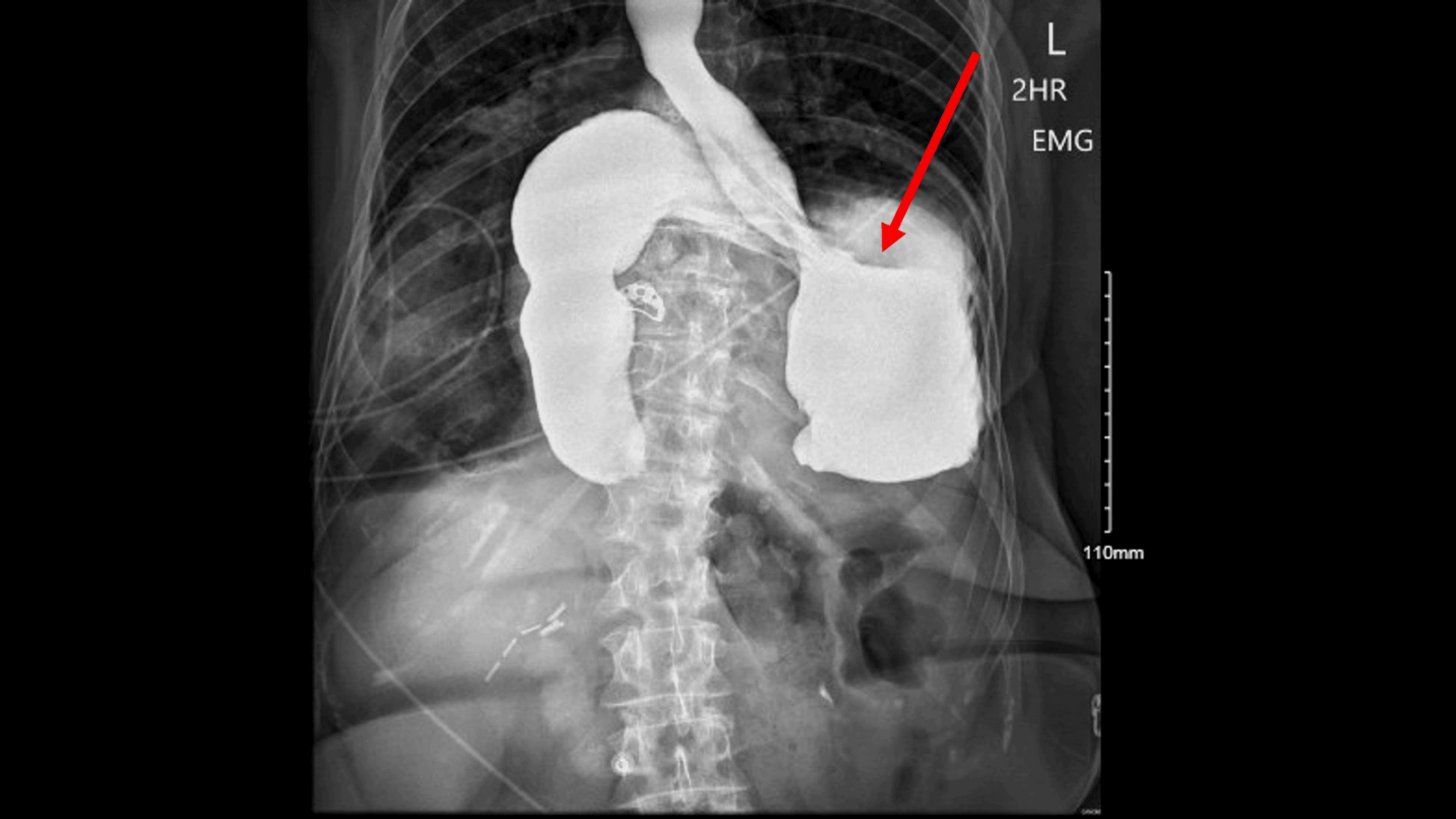
X-ray taken 7 days after readmission Findings: mesenteric axial volvulus with noted obstruction in the descending duodenum

A month later, the patient presented to the ED for nausea, vomiting, and abdominal pain. CT redemonstrated a recurrent, large hiatal hernia including the stomach, small bowel loops, transverse colon, and some surrounding ascites (Figure 5). Six days later, a CT further found part of the pancreas and colon compressing the left atrium, and small bilateral pleural effusions. Seven days after admission, an X-ray demonstrated mesenteric axial volvulus with noted obstruction in the descending duodenum (Figure 6). Eight days after admission, surgeons attempted to detorse the volvulus endoscopically but were unsuccessful. A new NG tube was placed for gastric decompression. Nine days after readmission, an open paraesophageal hernia repair (with Phasix™ mesh), gastric detorsion, gastropexy, and repair of gastric serosal tears were performed. The hospital course was complicated by postoperative ileus, which was resolving at the time of discharge. The patient was discharged 16 days after admission.

**Figure 7:**
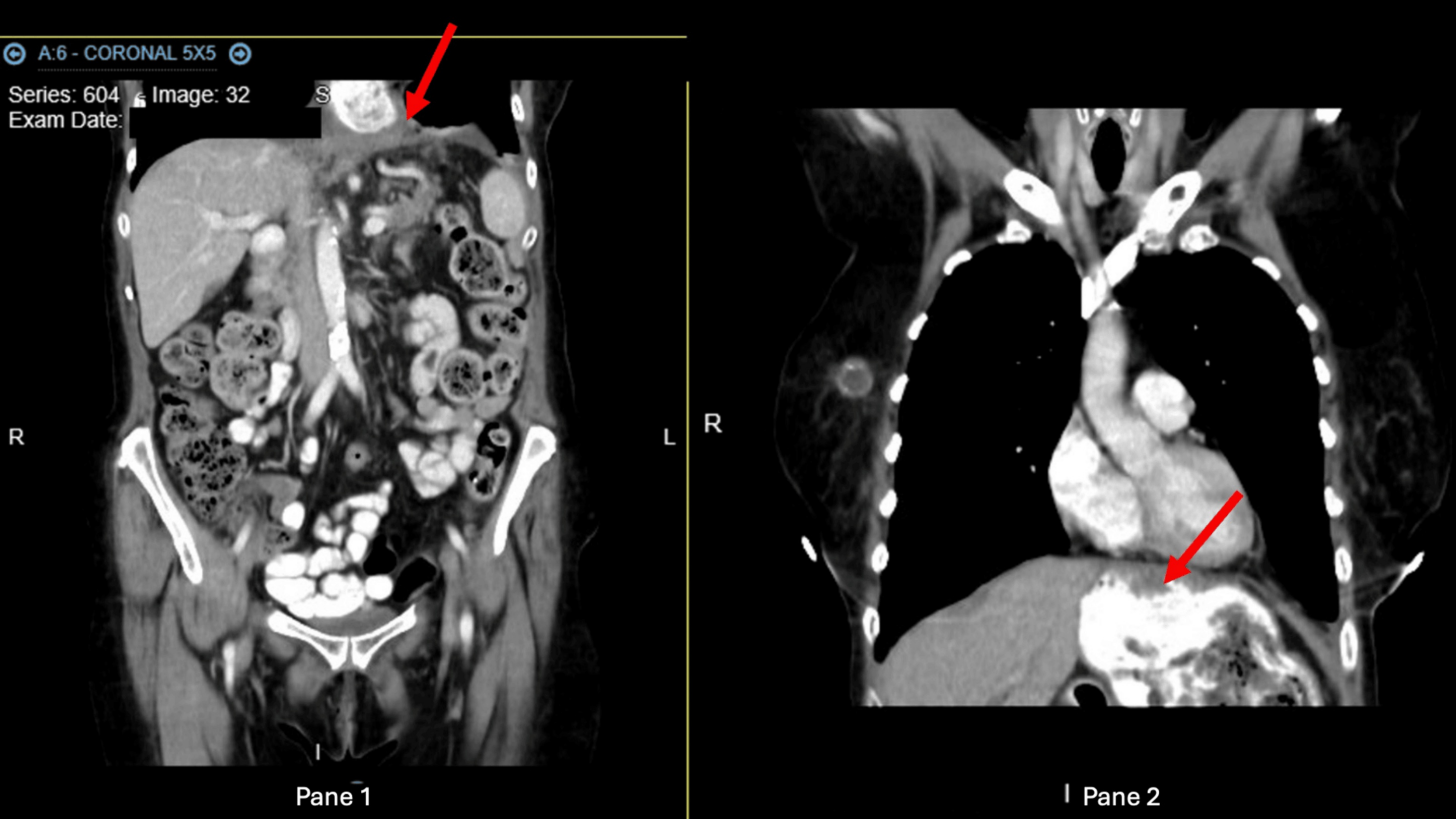
CT scan taken 113 days after open paraesophageal repair Findings: moderate hiatal hernia and moderate left pleural effusion CT: computed tomography

Unfortunately, 113 days after the open paraesophageal hernia repair, the patient returned with similar complaints of nausea, abdominal pain, and unintentional weight loss. A CT scan was performed, indicating a moderate hiatal hernia and moderate left pleural effusion (Figure 7). The patient presented with complaints of early satiety and nausea, which was confirmed via a gastric-emptying study demonstrating severe gastroparesis. 

**Figure 8:**
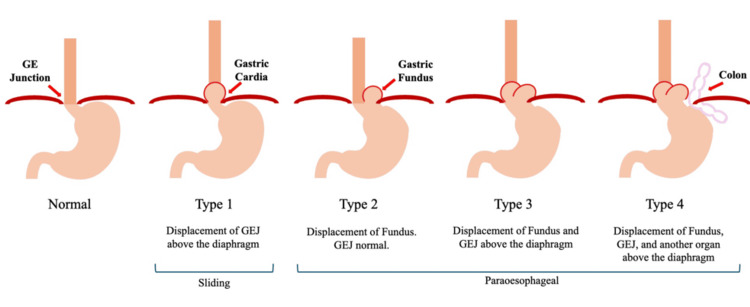
Types of hiatal hernias Image created by the authors

Figure 8 illustrates the different types of hiatal hernias in a pictorial format.

## Discussion

Paraesophageal hiatal hernias represent a distinct subset of hiatal hernias and are frequently associated with complications such as obstruction, strangulation, and gastric volvulus. Symptomatic cases are generally recommended for operative repair. Pancreatic herniation, in particular, is a rare manifestation. Only 16 cases of pancreatic herniation resulting in pancreatitis have been reported [3,4]. Patients with herniation, including involvement of the pancreas, are typically symptomatic, and all symptomatic paraesophageal hernias are recommended for repair, particularly those with volvulus. The introduction of robotic-assisted surgery has provided better visualization and articulation. Laparoscopic paraesophageal hernia repair has been noted to have high recurrence rates. Suggested factors have ranged from crural failure, failure to mobilize adequate esophageal length, or factors such as individual patient anatomy and physiology [5,6].

Optimizing the success of a paraesophageal hernia repair includes consideration of fundoplication, mesh reinforcement, and gastropexy. A meta-analysis investigating the role of fundoplication in paraesophageal hernia repair found that 69% of the 8600 enrolled patients underwent fundoplication. Those who underwent fundoplication had a lower risk of postoperative gastroesophageal reflux disease (GERD) and recurrence. However, they also found an increased risk of dysphagia in these patients [7]. In another recent meta-analysis, Weiss et al. investigated the role of fundoplication with and without mesh and found that there were no statistically significant differences between the two techniques. This finding remained consistent when comparing mesh vs. no mesh, absorbable vs. non-absorbable mesh, and with vs. without fundoplication. Overall, the rate of recurrence was 19.3%. Although not statistically significant, non-absorbable mesh with fundoplication may reduce recurrence and dysphagia, but absorbable mesh was associated with fewer complications. Primary suture repair had the highest rate of reoperation when compared to those who used mesh [8].

The role of gastropexy is also currently inconclusive, and it may be used as an adjunct to primary repair. Gastropexy appears to yield more promising results in emergent surgical repairs complicated by acute gastric volvulus and has been suggested to reduce recurrence rates. If gastropexy is performed in the absence of mesh placement or fundoplication, the complications associated with the latter two can be avoided. Overall, the evidence suggests that gastropexy can be a safe adjunct in selected patient populations, such as elderly patients or in emergent settings [9-11].

## Conclusions

Type IV paraesophageal hernia repair is a complicated surgery and is associated with high recurrence rates. Our patient’s gastroparesis could be a result of multiple surgical interventions impeding vagal nerve conduction or causing direct vagus nerve injury. The clinical course of this elderly patient highlights the need for innovation and investigation of new surgical protocols for these complicated hiatal hernias.

## References

[1] (2025). Gastrointestinal Surgical Emergencies. https://www.facs.org/media/o1gazfwy/2021_ms_gisemanual_final_01_22.pdf.

[2] Siegal SR, Dolan JP, Hunter JG (2017). Modern diagnosis and treatment of hiatal hernias. Langenbecks Arch Surg.

[3] (2026). SAGES - Society of American Gastrointestinal and Endoscopic Surgeons. Guidelines for the Surgical Treatment of Hiatal Hernias. SAGES. Published October 7.

[4] Coughlin M, Fanous M, Velanovich V (2011). Herniated pancreatic body within a paraesophageal hernia. World J Gastrointest Surg.

[5] Dimou FM, Velanovich V (2024). Dynamics of hiatal hernia recurrence: how important is a composite crural repair?. Hernia.

[6] Oliver MJ, Wilson AR, Kapila L (1990). Acute pancreatitis and gastric volvulus occurring in a congenital diaphragmatic hernia. J Pediatr Surg.

[7] Clapp B, Hamdan M, Mandania R (2022). Is fundoplication necessary after paraesophageal hernia repair? A meta-analysis and systematic review. Surg Endosc.

[8] Weiss BP, Emile SH, Horesh N (2025). Evaluating surgical outcomes of hiatal hernia repair techniques with and without fundoplication: a network meta-analysis. Surg Endosc.

[9] Alasmar M, McKechnie I, Chaparala RP (2023). Emergency surgery for hiatus hernias: does technique affect outcomes? A single-centre experience. Updates Surg.

[10] Cuschieri RJ, Wilson WA (1981). Incarcerated Bochdalek hernia presenting as acute pancreatitis. Br J Surg.

[11] Kafka NJ, Leitman IM, Tromba J (1994). Acute pancreatitis secondary to incarcerated paraesophageal hernia. Surgery.

